# Case report: Revealing a special and rare autoimmune GFAP astrocytopathy in the spinal cord succeeding Neurobrucellosis infection

**DOI:** 10.3389/fimmu.2022.950522

**Published:** 2022-08-05

**Authors:** Qiang He, Junxian Liu, Zehua Zhu, Yongxiang Tang, Lili Long, Kai Hu

**Affiliations:** ^1^ Department of Neurology, Xiangya Hospital, Central South University, Changsha, China; ^2^ National Clinical Research Center for Geriatric Disorders, Xiangya Hospital, Central South University, Changsha, China; ^3^ Clinical Research Center for Epileptic Disease of Hunan Province, Central South University, Changsha, China; ^4^ Department of Neurology, The Second Xiangya Hospital, Central South University, Changsha, China; ^5^ Department of Nuclear Medicine, Xiangya Hospital, Central South University, Changsha, China

**Keywords:** case report, glial fibrillary acidic protein, GFAP astrocytopathy, neurobrucellosis, FDG PET

## Abstract

Brucellosis, a zoonosis, can cause an inflammatory response in most organs and continues to be a public health problem in some endemic areas, whereas neurobrucellosis is a morbid form of brucellosis that affects the central nervous system (CNS) with poor prognosis. Autoimmune glial fibrillary acidic protein (GFAP) astrocytopathy is an autoimmune disease, and there have been no reports of a *Brucella* infection, leading to GFAP astrocytopathy. We report the case of a patient with a positive and high level of GFAP antibodies in the cerebrospinal fluid (CSF), following a *Brucella* infection. Although this patient did not show any responsible lesions in the diffusion sequence of the magnetic resonant imaging (MRI) scan, we found an evidence of thoracolumbar (T12) involvement on fluorodeoxyglucose (FDG) positron emission tomography (PET). The symptoms of spinal cord involvement were only partly relieved after initial treatment [doxycycline (0.1 g Bid) and rifampicin (0.6 g Qd) for 6 weeks]; however, they markedly improved after the subsequent immunosuppressive therapy [intravenous methylprednisolone (1,000 mg for 3 days)], followed by a 50% reduction from the preceding dose after 3 days, and subsequently, oral prednisone tablets (60 mg/day) was started, which was then gradually tapered [reduced to 10 mg/day every 1–2 weeks)]. The positive response to immunosuppressive therapy and treatment outcome strongly indicated the presence of an autoimmune neurological disease probably triggered by some infectious factors. Therefore, our findings reveal that a *Brucella* infection is one of the causes of autoimmune GFAP astrocytopathy, and when this infection is difficult to be identified by regular MRI, FDG PET can be used as a supplementary method for diagnosis and treatment.

## Introduction

Brucellosis is a zoonotic disease widely distributed around the world, which mainly infects the body from the skin, mucous membranes (of the respiratory tract, eye conjunctiva, and sexual organs), and digestive tract. It is an infection-led inflammatory disease, and the inflammatory response can be identified in most organs presenting clinical manifestations after infections such as endocarditis, arthritis, meningitis, joint lymphocytes, mononuclear cell infiltration, orchitis, nephritis, and liver granuloma (1). Like other manifestations of brucellosis, neurobrucellosis, a special form of brucellosis affecting the nervous system, also presents with signs and symptoms of inflammation. It primarily affects the central nervous system (CNS) and has a poor prognosis (2). Neurobrucellosis can manifest as meningitis, encephalitis, meningoencephalitis, meningeal vascular disease, brain abscess, demyelinating syndrome, and myelitis. Both encephalitis and myelitis are caused by bacteria that are present in the brain tissue and spinal cord. Moreover, reactive microgliosis and astrogliosis are involved in inflammatory responses. A *Brucella* infection and replication in the microglia, astrocytes, and brain endothelial cells lead to chronic infection in some patients, whereas the atypical clinical manifestations of neurobrucellosis can lead to misdiagnoses (3–5).

Autoimmune glial fibrillary acidic protein (GFAP) astrocytopathy is an inflammatory disease of the CNS. A specific immunoglobulin G (IgG) in the CSF that reacts with the intermediate filament protein GFAP in the cytoplasm of astrocytes is used as a biomarker. Clinical manifestations of the disease include fever, headache, encephalopathy, involuntary movements, myelitis, and abnormal vision (6, 7). The lesions often involve the subcortical white matter, basal ganglia, hypothalamus, brainstem, cerebellum, and spinal cord. A typical MRI feature is radial gadolinium enhancement perpendicular to the ventricles around the vessels of the brain in the white matter (8). Autoimmune GFAP astrocytopathy usually responds well to corticosteroids, although, in rare cases, long-term immunosuppression is required to alleviate the relapse course (6). Currently, there are no unified diagnostic criteria or consensus for autoimmune GFAP astrocytopathy, and it remains unclear how antibodies against GFAP are produced and whether they are involved in disease progression. Infectious pathogens may contribute to one of the major causes of GFAP astrocytopathy as triggering factors, although the underlying mechanisms still need to be clarified.

Here, we report the case of a patient who was infected with *Brucella* and tested positive for the GFAP antibody in the CSF. The symptoms of myelitis appeared throughout the disease phase, and initial antibacterial and successive immunosuppressive therapy relieved the symptoms in steps. Concurrently, concerns related to the diagnosis and treatment are discussed in detail, and potential relationships between the two diseases are explored.

## Case representation

A 42-year-old man was admitted to our hospital with a chief complaint of fever for 1 month and bilateral lower extremity numbness, weakness, and difficulty urinating for 10 days. One month prior, the patient had developed fever, chills, and headache with no obvious cause and his temperature was up to 39.4°C. Cefixime was administered for 1 week at a local clinic. However, the patient’s symptoms did not improve. The patient was then transferred to another local hospital. There were no positive findings in blood, urine, or stool cultures, among others. Ten days later, he developed a numbness and weakness of both lower limbs, unsteady walking, difficulty urinating, hearing loss in the left ear, and blurred vision. At the local the Centers for Disease Control, the serum agglutination test was positive for brucellosis at a titer of 1:100 (++), and the Rose Bengal Plate Test (RBPT) was positive (+). With the diagnosis of brucellosis, he was prescribed doxycycline (0.1 g Bid) and rifampicin (0.6 g Qd). His fever symptom was gradually relieved, but the weakness and numbness of the lower extremities and difficulty in urinating did not improve. The patient was admitted to our hospital for further treatment. This patient had a history of hypertension, coronary heart disease, and urticaria in the past. There was no previous history of infection or vaccination before the disease onset. His personal and family histories were unremarkable.

**Figure 1:**
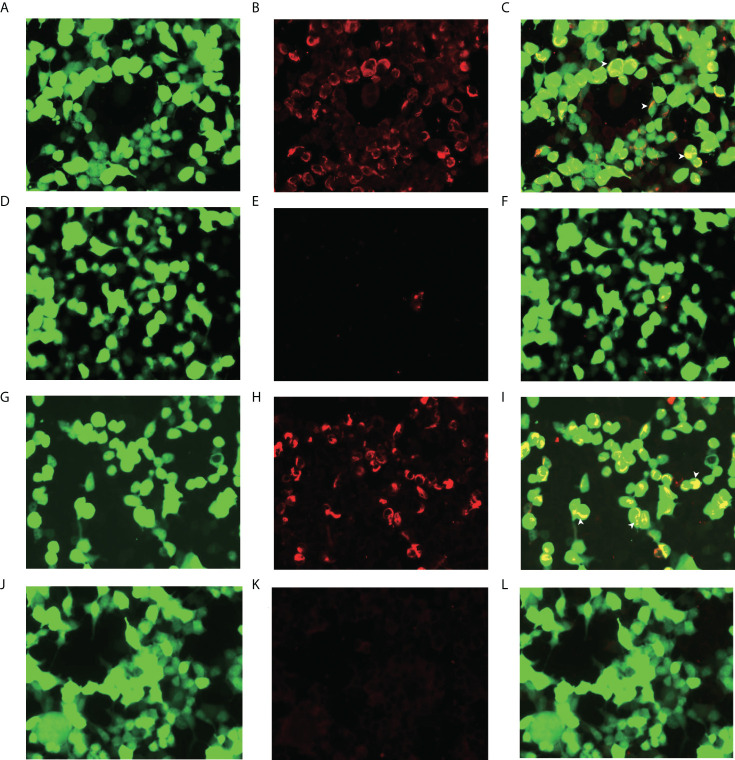
Glial fibrillary acidic protein (GFAP-IgG) by GFAP-transfected cell-based immunofluorescence assay. Cells were expressing green fluorescent protein-tagged GFAP (green) and immunostained (red if positive). **(A, B, C)** Examination of cerebrospinal fluid (CSF) in first admission. **(D, E, F)** Examination of serum in first admission. **(G, H, I)** Examination of CSF in second admission. **(J, K, L)** Examination of serum in second admission. **(C, F, I, L)** Merged images revealed the colocalization of the GFAP antibody and astrocyte (white arrows) (scale bar=50 μm).

**Figure 2:**
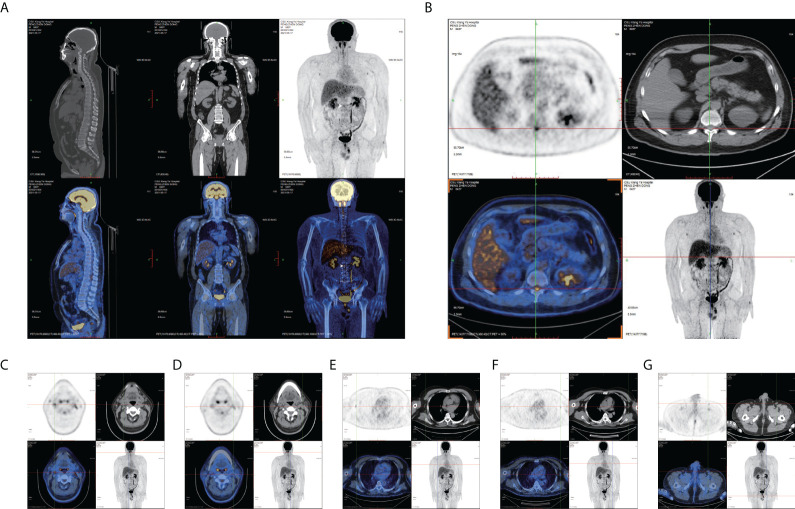
FDG PET images. **(A)** Sagittal and coronal PET/CT show a high concentration in the thoracolumbar segment (white arrow). **(B)** Axial PET/CT shows an extremely abnormal hypermetabolism of FDG near the T12 thoracolumbar segment (maximum standardized uptake value: 6.321). Axial PET/CT in **(C, D)** bilateral cervical lymph nodes, **(E, F)** bilateral axillary lymph nodes, and **(G)** epididymis.

The examination revealed significant findings for both lower limbs with grade 4+ muscle power. Thermal and pain sensations diminished below the thoracic T11 level with an obvious tightness. The cremasteric reflex was not elicited. Romberg’s sign was positive. The lymph node color Doppler ultrasound showed multiple lymphadenectasis in the bilateral axilla, groin, and right epididymis. The electromyogram and MRI of the head and spinal cord yielded normal results. In CSF, the white blood cell count was slightly increased, and lymphocytes and plasma cells were visible and were mainly observed. These findings suggested an inflammatory reaction. High titers of GFAP IgG antibodies (1:32) were detected in the CSF but not in the serum (**Figures 1A–F**). Tests for other autoantibodies in the CSF and serum, including AQP4-IgG, MBP-IgG, and MOG-IgG, were negative. Positive MRI evidence was absent, although symptoms and physical examination suggested spinal cord involvement. FDG PET was performed to identify possible lesions in the spinal cord and other parts of the nervous system. The abnormal concentration of FDG was mainly found in the thoracolumbar segment of the spinal cord, and the hypermetabolism region was located near the thoracic T12 level. The maximum standard uptake value (SUV_max_) was 6.321, which was significantly higher than that in the normal area of the spinal cord (**Figures 2A, B**). FDG accumulation also occurred at other sites, such as the bilateral cervical lymph nodes, bilateral axillary lymph nodes, thoracoabdominal pelvic lymph nodes, and epididymis (**Figures 2C–G**).

Infective myelitis caused by neurobrucellosis is predominant in the initial stages of the disease, with coexisting autoimmune GFAP astrocytopathy. It is not reasonable to administer high-dose steroid pulse treatment in the case of the brucellosis infection spreading to multiple systems in the body. Therefore, plasma exchange and intravenous immunogloblin combined with low-dose steroid therapy (prednisone tablets 30 mg Qd) were administered. Meanwhile, a first-line anti-infection regimen [doxycycline (0.1 g Bid) and rifampicin (0.6 g Qd)] and ceftriaxone (2 g Qd) were also added. The patient’s numbness and weakness in the lower extremities did not worsen. Moreover, after plasma exchange, his symptoms stabilized, with a very slight trend toward alleviation. The patient was maintained on doxycycline, rifampicin, and prednisone after discharge from the hospital. However, the numbness and weakness of both lower extremities and waist tightness persisted. Difficulty in urinating occasionally occurs. The patient was readmitted to the hospital 1 month after discharge for further treatment. At readmission, all the brucellosis-related laboratory tests were negative. Spinal MRI findings were unremarkable. Higher titers of GFAP IgG antibodies (1:32) were detected in the CSF (**Figures 1G–L**). An elevated protein level of 89 mg/L (normal range: 10.0–30.0 mg/L) and oligoclonal bands (OCBs) (more than two bands) were detected in the CSF but not in the serum. In the second stage of the disease, autoimmune GFAP astrocytopathy occupied the predominant position and was responsible for the maintenance of symptoms and signs of the spinal cord, since the initial neurobrucellosis disappeared after first-line treatment with anti-infection therapy. Therefore, the patient was treated with intravenous methylprednisolone (1,000 mg for 3 days), followed by a 50% reduction of the dose after 3 days, followed by oral prednisone tablets (60 mg/day), were then gradually tapered (reduced to 10 mg/day every 1–2 weeks). All symptoms improved after high-dose steroid and oral tablet therapies.

## Discussion

In the present case, the patient was diagnosed with autoimmune GFAP astrocytopathy secondary to a *Brucella* infection. However, the occurrence of autoimmune disease after a *Brucella* infection is comparatively rare; therefore, the identification and diagnosis of GFAP astrocytopathy are not as easy as expected. Our patient had persistent spinal cord involvement, presenting symptoms such as the numbness and weakness of both lower extremities as well as difficulty in urination. The patient’s first condition improved slightly after anti-infection therapy, but the symptoms and signs of spinal cord involvement remained unresolved. Elevated levels of GFAP antibodies in the patient’s CSF were confirmed using laboratory tests. Although the MRI scan did not show corresponding lesions, FDG PET demonstrated extremely abnormal hypermetabolism, indicating an infectious condition in the spinal cord and other body parts. As a result, anti-infection therapy, plasma exchange, IVIG, and low-dose hormone therapy were administered in the first stage. It is believed that the myelitis in the early stage was mainly attributed to a *Brucella* infection while in the later stage, myelitis was predominantly caused by autoimmune GFAP astrocytopathy. After one course of high-dose hormone methylprednisolone percussion therapy in the later stage, the numbness and weakness of both lower extremities, waist tightness, and labored urination were all ameliorated, which further confirmed the later predominance of GFAP astrocytopathy. Although the symptoms of our patient and the positive response to treatment established the diagnosis, unfortunately, the patient lacked complete epidemiological investigations and pathological examination. Six months after being discharged from the hospital, we followed up the patient by telephone and found that all the symptoms of the patient continued to improve. Moreover, the patient adhered to taking the medication regularly, and no adverse and unanticipated events occurred during the treatment.

According to previous studies, GFAP antibodies can be found in the CSF after traumatic brain injury, certain tumors, viral infections (such as Herpes Simplex Virus), multiple sclerosis, diabetes, and idiopathic intracranial hypertension (9–14). There have been no reports of elevated levels of GFAP antibodies in the CSF after bacterial infection, and how GFAP antibodies are produced remains unclear. These signs led us to wonder why the intracellular protein GFAP causes an increase in autoantibodies. How does bacterial infection contribute to GFAP astrocytopathy? First of all, in the process of bacteria entering and egressing cells, it may cause the destruction of cell membranes and the release of cytoplasmic contents. This greatly increases the chance that the cytoplasmic contents will be falsely recognized by the immune system. Secondly, the infection of astrocytes and microglia with *Brucella* induces the secretion of proinflammatory mediators such as IL-6, IL-1β, and tumor necrosis factor (TNF)-α (15). On one hand, these proinflammatory mediators can lead to astrocytes apoptosis. During this period, GFAP in astrocytes has the potential to expose self-epitopes and may change its subcellular distribution through proteolytic cleavage, phosphorylation, or dephosphorylation (15–17). On the other hand, these proinflammatory mediators can disrupt the integrity of the blood–brain barrier and attract immune cells from the peripheral circulation into the CNS (15, 18). Moreover, consistent with the paraneoplastic origin of GFAP antibodies, it is unclear whether *Brucella* has antigens similar to GFAP, which causes an immune response after infection. Although the above evidence shows the possibility of the production of GFAP antibodies after a bacterial infection, some researchers still think that this specific IgG directed at intracellular antigens lacks pathogenicity to cell targets *in vivo*, and it is believed that the GFAP antibody can only be used as a marker for activated CD8+ cytotoxic T cells, whereas the real pathogenicity may be those unknown autoantibodies raised together with GFAP antibodies (7). As the exact mechanism is still debated, further experiments are required to confirm this.

In our case, it is also worth pointing out that we did not find responsible lesions in the MRI scan, but after using FDG PET as assistance, we identified the abnormal hypermetabolism of FDG near thoracic T12 thoracolumbar segment. According to previous cases, FDG metabolism can be extremely abnormal in infectious diseases, and the FDG uptake rate is higher than its counterparts in non-infectious chronic active inflammation. The maximum standardized uptake value (SUV_max_) of infectious diseases can be significantly increased (up to 5.0–7.0, or even higher), whereas non-infectious inflammation shows moderate increases (generally <5.0, or insignificant increase) (19–21). Therefore, the maximum standardized uptake value (SUV_max_) of the patient’s spinal cord lesion was 6.321, indicating that the spinal cord lesion was caused by a *Brucella* infection. Due to some economic factors, FDG PET was not performed again at the time of readmission. Changes in the functional metabolic phase of CNS infectious diseases generally present earlier than changes in the anatomical structure phase, and the FDG PET manifestations of tiny and occult lesions are much more evident than lesions with obvious abnormal morphological structures. FDG PET helps detect early lesions that do not form structural abnormalities on MRI or small lesions that cannot be identified by MRI. These lesions can be identified through the functional metabolic phase, and changes in the metabolic function can be reflected in time. The advantage of the functional metabolic imaging of FDG PET is that it can compensate for the deficiency of regular MRI scans, and it possesses a real application value in clinical diagnosis and treatment.

In conclusion, autoimmune GFAP astrocytopathy should be considered for patients diagnosed with neurobrucellosis, and the presence of GFAP astrocytopathy should be confirmed by the detection of GFAP antibodies in the CSF and serum. Immunosuppressive therapy should be administered at a proper time and in a proper manner. The FDG PET provides an optional auxiliary method for patients who have no obvious abnormalities on MRI scans. Prompt identification and effective treatment can reduce the incidence of critical events.

## Data availability statement

The original contributions presented in the study are included in the article/supplementary material. Further inquiries can be directed to the corresponding author.

## Ethics statement

The studies involving human participants were reviewed and approved by the Ethics Committee of Xiangya Hospital of Central South University. The patients/participants provided their written informed consent to participate in this study. Written informed consent was obtained from the patient for the publication of any potentially identifiable images or data included in the article.

## Author contributions

KH designed the study, reviewed, and revised the manuscript. QH collected and analyzed data. JL drafted the manuscript. ZZ, YT, and LL reviewed and revised the manuscript. All the authors read and approved the final manuscript.

## Funding

This study was supported by grants from the Hunan Provincial Development and Reform Investment Fund (No.2021-212 to KH), the Innovative construction foundation of Hunan province (grant number: 2021SK4001).

## Acknowledgments

We are grateful to our patient and his family for their support of this study.

## Conflict of interest

The authors declare that the research was conducted in the absence of any commercial or financial relationships that could be construed as a potential conflict of interest.

## Publisher’s note

All claims expressed in this article are solely those of the authors and do not necessarily represent those of their affiliated organizations, or those of the publisher, the editors and the reviewers. Any product that may be evaluated in this article, or claim that may be made by its manufacturer, is not guaranteed or endorsed by the publisher.
